# Complete genome sequence of *Staphylothermus hellenicus* P8^T^

**DOI:** 10.4056/sigs.2054696

**Published:** 2011-09-23

**Authors:** Iain Anderson, Reinhard Wirth, Susan Lucas, Alex Copeland, Alla Lapidus, Jan-Fang Cheng, Lynne Goodwin, Samuel Pitluck, Karen Davenport, John C. Detter, Cliff Han, Roxanne Tapia, Miriam Land, Loren Hauser, Amrita Pati, Natalia Mikhailova, Tanja Woyke, Hans-Peter Klenk, Nikos Kyrpides, Natalia Ivanova

**Affiliations:** 1 DOE Joint Genome Institute, Walnut Creek, California, USA; 2 University of Regensburg, Microbiology – Archaeenzentrum, Regensburg, Germany; 3 Los Alamos National Laboratory, Los Alamos, New Mexico, USA; 4 Biosciences Division, Oak Ridge National Laboratory, Oak Ridge, Tennessee, USA; 5 DSMZ – German Collection of Microorganisms and Cell Cultures GmbH, Braunschweig, Germany

**Keywords:** Archaea, *Crenarchaeota*, *Desulfurococcaceae*, hyperthermophile, hydrothermal vent, anaerobe

## Abstract

*Staphylothermus hellenicus* belongs to the order *Desulfurococcales* within the archaeal phylum *Crenarchaeota*. Strain P8^T^ is the type strain of the species and was isolated from a shallow hydrothermal vent system at Palaeochori Bay, Milos, Greece. It is a hyperthermophilic, anaerobic heterotroph. Here we describe the features of this organism together with the complete genome sequence and annotation. The 1,580,347 bp genome with its 1,668 protein-coding and 48 RNA genes was sequenced as part of a DOE Joint Genome Institute (JGI) Laboratory Sequencing Program (LSP) project.

## Introduction

**Figure 1:**
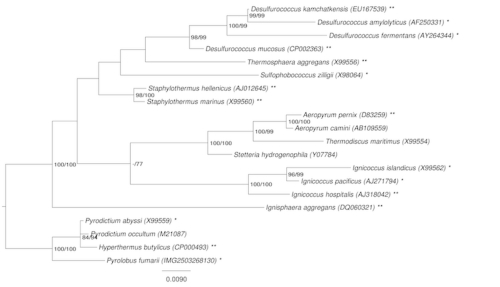
Phylogenetic tree highlighting the position of *S. hellenicus* relative to the type strains of the other species within the order *Desulfurococcales*. The tree was inferred from 1,333 aligned characters [4,5] of the 16S rRNA gene sequence under the maximum likelihood (ML) criterion [6]. Rooting was done initially using the midpoint method [7] and then checked for its agreement with the current classification (Table 1). The branches are scaled in terms of the expected number of substitutions per site. Numbers adjacent to the branches are support values from 1,000 ML bootstrap replicates [8] (left) and from 1,000 maximum parsimony bootstrap replicates [9] (right) if larger than 60%. Lineages with type strain genome sequencing projects registered in GOLD [10] are labeled with one asterisk, those listed as 'Complete and Published' with two asterisks.

Strain P8^T^ (=DSM 12710 = JCM 10830) is the type strain of the species *Staphylothermus hellenicus*. It was isolated from a shallow hydrothermal vent at Palaeochori Bay near the island of Milos, Greece [1]. There is one other validly named species in the genus, *S. marinus*, for which a complete genome sequence has been determined and published [2,3]. The *S. hellenicus* genome is the ninth to be published from the order *Desulfurococcales* in the phylum *Crenarchaeota*. The only other genus in the *Desulfurococcales* for which two species have been sequenced is *Desulfurococcus*. Figure 1 shows the phylogenetic position of *S. hellenicus* with respect to the other species in the order *Desulfurococcales*.

## Organism information

**Figure 2:**
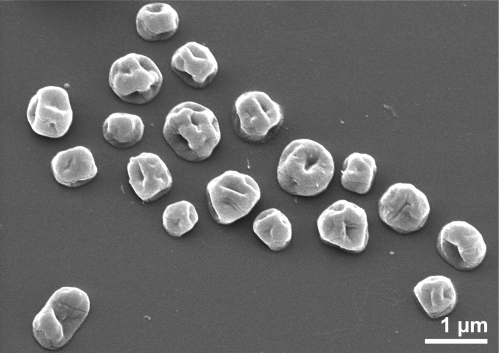
Scanning electron micrograph of *S. hellenicus* P8^T^.

*S. hellenicus* was isolated from sediment at Palaeochori Bay, Milos, Greece [1]. For isolation, 1 ml of sediment was added to half-strength SME medium [11] with 2% elemental sulfur and incubated at 90°C under H_2_/CO_2_. Colonies were isolated on plates with the same medium and with 1% Phytagel and 2-3% sodium alginate added [1]. *S. hellenicus* is a regular-shaped coccus (Figure 2) which can form large aggregates of up to fifty cells, similar to *S. marinus* [1,12]. No flagella were observed and cells were nonmotile. The temperature range for growth of *S. hellenicus* is 70-90°C, with an optimum at 85°C [1]. The salinity range was from 2% to 8% NaCl, and the optimum was 4% NaCl [1]. The pH range for growth was from 4.5 to 7.5. The optimum pH was 6.0 [1]. *S. hellenicus* is a strict anaerobe, and can grow under H_2_/CO_2_ or N_2_/CO_2_ [1]. It is a heterotroph which grows well on yeast extract but poorly on peptone [1]. Many carbon sources were tested, but no growth was observed, showing that a complex nutrient source is required [1]. Elemental sulfur was required for growth [1]. The features of the organism are listed in Table 1.

**Table 1:** Classification and general features of *S. hellenicus* P8^T^ according to the MIGS recommendations [13]

MIGS ID	Property	Term	Evidence code^a^
	Current classification	Domain *Archaea*	TAS [14]
		Phylum *Crenarchaeota*	TAS [15,16]
		Class *Thermoprotei*	TAS [16,17]
		Order *Desulfurococcales*	TAS [16,18]
		Family *Desulfurococcaceae*	TAS [19-21]
		Genus *Staphylothermus*	TAS [12,22]
		Species *Staphylothermus hellenicus*	TAS [1]
		Type strain P8	TAS [1]
	Cell shape	coccus	TAS [1]
	Motility	nonmotile	TAS [1]
	Sporulation	nonsporulating	NAS
	Temperature range	70-90°C	TAS [1]
	Optimum temperature	85°C	TAS [1]
MIGS-6.3	Salinity	2-8% NaCl (optimum 4%)	TAS [1]
MIGS-22	Oxygen requirement	anaerobe	TAS [1]
	Carbon source	yeast extract	TAS [1]
	Energy metabolism	heterotrophic	TAS [1]
MIGS-6	Habitat	marine geothemally heated areas	TAS [1]
MIGS-15	Biotic relationship	free-living	TAS [1]
MIGS-14	Pathogenicity	none	NAS
	Biosafety level	1	NAS
	Isolation	geothermally heated sediment	TAS [1]
MIGS-4	Geographic location	Palaeochori Bay, Milos, Greece	TAS [1]
MIGS-5	Isolation time	September 1996	TAS [1]
MIGS-4.1 MIGS-4.2	Latitude longitude	36.674 24.517	TAS [1]
MIGS-4.3	Depth	4-10 m	TAS [1]
MIGS-4.4	Altitude	not applicable	

a) Evidence codes - IDA: Inferred from Direct Assay; TAS: Traceable Author Statement (i.e., a direct report exists in the literature); NAS: Non-traceable Author Statement (i.e., not directly observed for the living, isolated sample, but based on a generally accepted property for the species, or anecdotal evidence). These evidence codes are from the Gene Ontology project [23].

## Genome sequencing information

### Genome project history

This organism was selected for sequencing on the basis of its phylogenetic position and is part of a Laboratory Sequencing Project (LSP) to sequence diverse archaea. The genome project is listed in the Genomes On Line Database [10] and the complete genome sequence has been deposited in GenBank. Sequencing, finishing, and annotation were performed by the DOE Joint Genome Institute (JGI). A summary of the project information is shown in Table 2.

**Table 2:** Genome sequencing project information

**MIGS ID**	**Property**	**Term**
MIGS-31	Finishing quality	Finished
MIGS-28	Libraries used	Illumina standard library, 454 standard library, 454 28 kb paired end library
MIGS-29	Sequencing platforms	Illumina GA II, 454 GS FLX Titanium
MIGS-31.2	Sequencing coverage	462× with Illumina, 132× with 454
MIGS-30	Assemblers	Velvet, Newbler, phrap
MIGS-32	Gene calling method	Prodigal, GenePRIMP
	INSDC ID	CP002051
	Genbank Date of Release	June 1, 2010
	GOLD ID	Gc01350
	NCBI project ID	33683
MIGS-13	Source material identifier	DSM 12710
	Project relevance	Phylogenetic diversity, biotechnology

### Growth conditions and DNA isolation

*S. hellenicus* P8^T^ cells were grown in a 300 liter fermenter at 85°C in SME medium [11] with 0.1% yeast extract, 0.1% peptone, and 0.7% elemental sulfur under a 200 kPa N_2_ atmosphere. DNA was isolated with a Qiagen Genomic 500 DNA Kit.

### Genome sequencing and assembly

The genome of *S. hellenicus* was sequenced at the Joint Genome Institute (JGI) using a combination of Illumina and 454 technologies. An Illumina GA II shotgun library with reads of 730 Mb, a 454 Titanium draft library with average read length of 310.5 +/- 187.8 bases, and a paired end 454 library with an average insert size of 28 Kb were generated for this genome. Illumina sequencing data was assembled with Velvet [24], and the consensus sequences were shredded into 1.5 kb overlapped fake reads and assembled together with the 454 data with Newbler. Draft assemblies were based on 208 Mb 454 draft data.

The initial Newbler assembly contained 4 contigs in 1 scaffold. We converted the initial 454 assembly into a phrap assembly by making fake reads from the consensus, collecting the read pairs in the 454 paired end library. The Phred/Phrap/Consed software package was used for sequence assembly and quality assessment [25-27] in the following finishing process. After the shotgun stage, reads were assembled with parallel phrap (High Performance Software, LLC). Possible mis-assemblies were corrected with gapResolution (Cliff Han, unpublished), Dupfinisher [28], or sequencing cloned bridging PCR fragments with subcloning or transposon bombing (Epicentre Biotechnologies, Madison, WI). Gaps between contigs were closed by editing in Consed, by PCR and by Bubble PCR primer walks. A total of 23 additional reactions were necessary to close gaps and to raise the quality of the finished sequence.

### Genome annotation

Genes were identified using Prodigal [29], followed by a round of manual curation using GenePRIMP [30]. The predicted CDSs were translated and used to search the National Center for Biotechnology Information (NCBI) nonredundant database, UniProt, TIGRFam, Pfam, PRIAM, KEGG, COG, and InterPro databases. The tRNAScanSE tool [31] was used to find tRNA genes, whereas ribosomal RNAs were found by using BLASTn against the ribosomal RNA databases. The RNA components of the protein secretion complex and the RNase P were identified by searching the genome for the corresponding Rfam profiles using INFERNAL [32]. Additional gene prediction analysis and manual functional annotation was performed within the Integrated Microbial Genomes (IMG) platform [33] developed by the Joint Genome Institute, Walnut Creek, CA, USA [34].

## Genome properties

**Figure 3:**
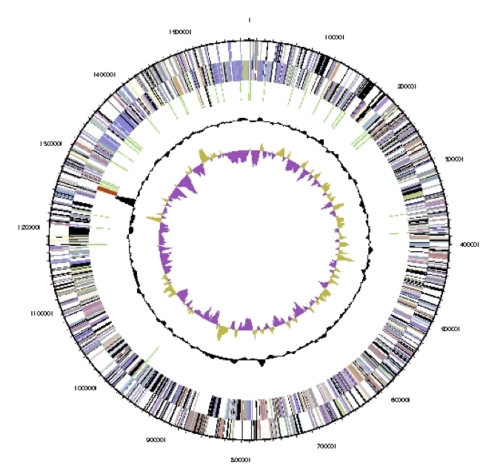
Graphical circular map of the chromosome. From outside to the center: Genes on forward strand (colored by COG categories), genes on reverse strand (colored by COG categories), RNA genes (tRNAs green, rRNAs red, other RNAs black), GC content, and GC skew.

The genome includes one chromosome and no plasmids, for a total size of 1,580,437 bp (Table 3 and Figure 3). This genome size is close to the average for *Desulfurococcales*. The GC percentage is 36.8%, which is lower than most of the *Desulfurococcales*. A total of 1,716 genes were identified: 48 RNA genes and 1,668 protein-coding genes. There are 69 pseudogenes, comprising 4.1% of the protein-coding genes. About 62% of predicted genes begin with ATG, 30% begin with TTG, and 7% begin with GTG. There is one copy of each ribosomal RNA. Table 4 shows the distribution of genes in COG categories.

**Table 3:** Nucleotide content and gene count levels of the genome

Attribute	Value	% of total^a^
Size (bp)	1,580,437	100.0%
G+C content (bp)	582,173	36.8%
Coding region (bp)	1,383,053	87.5%
Number of replicons	1	
Extrachromosomal elements	0	
Total genes	1,716	
RNA genes	48	
rRNA operons	1	
Protein-coding genes	1,668	100.0%
Pseudogenes	69	4.1%
Genes with function prediction	975	58.5%
Genes in paralog clusters	98	5.9%
Genes assigned to COGs	1,093	65.5%
Genes assigned Pfam domains	1,135	68.0%
Genes with signal peptides	129	7.7%
Genes with transmembrane helices	342	20.5%
CRISPR repeats	3	% of total^a^

a) The total is based on either the size of the genome in base pairs or the total number of protein coding genes in the annotated genome

**Table 4:** Number of genes associated with the 25 general COG functional categories

**Code**	**Value**	**%age**^a^	**Description**
J	161	9.7	Translation
A	2	0.1	RNA processing and modification
K	59	3.5	Transcription
L	72	4.3	Replication, recombination and repair
B	2	0.1	Chromatin structure and dynamics
D	7	0.4	Cell cycle control, mitosis and meiosis
Y	0	0.0	Nuclear structure
V	18	1.1	Defense mechanisms
T	20	1.2	Signal transduction mechanisms
M	39	2.3	Cell wall/membrane biogenesis
N	4	0.2	Cell motility
Z	0	0.0	Cytoskeleton
W	0	0.0	Extracellular structures
U	11	0.7	Intracellular trafficking and secretion
O	49	2.9	Posttranslational modification, protein turnover, chaperones
C	79	4.7	Energy production and conversion
G	79	4.7	Carbohydrate transport and metabolism
E	73	4.4	Amino acid transport and metabolism
F	44	2.6	Nucleotide transport and metabolism
H	53	3.2	Coenzyme transport and metabolism
I	15	0.9	Lipid transport and metabolism
P	67	4.0	Inorganic ion transport and metabolism
Q	5	0.3	Secondary metabolites biosynthesis, transport and catabolism
R	194	11.6	General function prediction only
S	116	7.0	Function unknown
-	575	34.5	Not in COGs

a) The total is based on the total number of protein coding genes in the annotated genome.

## Comparison with the *S. marinus* genome

The genome of *S. hellenicus* is slightly larger than the genome of *S. marinus* (1.58 Mbp vs. 1.57 Mbp), and the number of protein-coding genes is also larger (1668 vs. 1610). However, the number of pseudogenes is also higher in *S. hellenicus* (69 vs. 40). Some of the COG categories show different numbers of genes between the two organisms. *S. hellenicus* has 25 additional genes that do not belong to COGs. *S. hellenicus* has greater numbers of genes involved in cell wall biogenesis (39 vs. 23), nucleotide transport and metabolism (44 vs. 39) and carbohydrate transport and metabolism (79 vs. 72), while *S. marinus* has greater numbers of genes in the categories of energy production and conversion (92 vs. 79) and inorganic ion transport and metabolism (85 vs. 67).

The genes involved in cell wall metabolism that are in *S. hellenicus* but not in *S. marinus* are genes involved in nucleotide-sugar metabolism and glycosyltransferases, suggesting that *S. hellenicus* may have a greater variety of sugars attached to glycolipids and glycoproteins. Most of the additional *S. hellenicus* genes are located within a region of fifty genes on the chromosome (Shell_0865-Shell_0915) that is not present in *S. marinus*. The additional genes in *S. hellenicus* involved in nucleotide metabolism include adenylosuccinate synthase, adenylosuccinate lyase, and GMP synthase. Both *S. hellenicus* and *S. marinus* lack *de novo* purine synthesis, but the presence of these three additional enzymes suggests that *S. hellenicus* may be able to synthesize AMP and GMP from IMP, while *S. marinus* is unable to do so. The additional genes in carbohydrate transport and metabolism include nucleotide-sugar modifying enzymes that were also included in cell wall metabolism, but they also include a probable β-1,4-endoglucanase (cellulase) from glycosyl hydrolase family 5.

The genes found in *S. marinus* but not in *S. hellenicus* belong to the categories of energy production and conversion, and inorganic ion transport and metabolism. They include proteins related to subunits of multisubunit cation:proton antiporters and proteins related to subunits of NADH dehydrogenase and formate hydrogen lyase. These proteins are similar to subunits of mbh, a multisubunit membrane-bound hydrogenase from *Pyrococcus furiosus* [35], and mbx, a multisubunit complex of unknown function that probably has a role in sulfur reduction, also from *P. furiosus* [36]. *S. marinus* has three operons related to mbh and mbx, while *S. hellenicus* has only one, suggesting that the three operons may be redundant in function in *S. marinus*. Since *S. marinus* and *S. hellenicus* lack other enzymes involved in sulfur reduction, it is possible that these mbh/mbx-related operons play a role in sulfur reduction in these organisms.
